# Prolonged sedentary time and physical activity in workplace and non-work contexts: a cross-sectional study of office, customer service and call centre employees

**DOI:** 10.1186/1479-5868-9-128

**Published:** 2012-10-26

**Authors:** Alicia A Thorp, Genevieve N Healy, Elisabeth Winkler, Bronwyn K Clark, Paul A Gardiner, Neville Owen, David W Dunstan

**Affiliations:** 1 Baker IDI Heart and Diabetes Institute, Level 4 The Alfred Centre, 99 Commercial Road, Melbourne, Victoria 3004, Australia; 2 School of Population Health, Cancer Prevention Research Centre, The University of Queensland, Brisbane, Level 3, Public Health Building, Herston Road, Herston, Queensland 4006, Australia; 3 School of Exercise and Nutrition Sciences, Deakin University, 221 Burwood Highway, Burwood, Victoria, 3125, Australia; 4 Edith Cowan University Health and Wellness Institute, Edith Cowan University, 270 Joondalup Drive, Joondalup, Western Australia, 6027, Australia; 5 Department of Epidemiology and Preventive Medicine, Monash University, Level 6 The Alfred Centre, 99 Commercial Road, Melbourne, Victoria, 3004, Australia; 6 Translating Research Into Practice (TRIP) Centre, Mater Medical Research Institute, Level 3, Aubigny Place, Mater Hospitals, South Brisbane, QLD, 4101, Australia

**Keywords:** Occupational sitting, Active time, Workers, Leisure-time

## Abstract

**Background:**

To examine sedentary time, prolonged sedentary bouts and physical activity in Australian employees from different workplace settings, within work and non-work contexts.

**Methods:**

A convenience sample of 193 employees working in offices (131), call centres (36) and customer service (26) was recruited. Actigraph GT1M accelerometers were used to derive percentages of time spent sedentary (<100 counts per minute; cpm), in prolonged sedentary bouts (≥20 minutes or ≥30 minutes), light-intensity activity (100–1951 cpm) and moderate-to-vigorous physical activity (MVPA; ≥1952 cpm). Using mixed models adjusted for confounders, these were compared for: work days versus non-work days; work hours versus non-work hours (work days only); and, across workplace settings.

**Results:**

Working hours were mostly spent sedentary (77.0%, 95%CI: 76.3, 77.6), with approximately half of this time accumulated in prolonged bouts of 20 minutes or more. There were significant (p<0.05) differences in all outcomes between workdays and non-work days, and, on workdays, between work- versus non-work hours. Results consistently showed “work” was more sedentary and had less light-intensity activity, than “non-work”. The period immediately after work appeared important for MVPA. There were significant (p<0.05) differences in all sedentary and activity outcomes occurring during work hours across the workplace settings. Call-centre workers were generally the most sedentary and least physically active at work; customer service workers were typically the least sedentary and the most active at work.

**Conclusion:**

The workplace is a key setting for prolonged sedentary time, especially for some occupational groups, and the potential health risk burden attached requires investigation. Future workplace regulations and health promotion initiatives for sedentary occupations to reduce prolonged sitting time should be considered.

## Background

There is emerging evidence that time spent in sedentary behaviour (defined as any waking behaviour characterized by an energy expenditure ≤1.5 METs while in a sitting or reclining posture) [1] is deleteriously associated with all-cause and cardiovascular disease mortality [2-4] and with biomarkers of cardio-metabolic risk [5-7]. These associations are largely independent of time spent in moderate-to-vigorous physical activity (MVPA) [7,8]. Most of the evidence is from population-based studies that have focussed on sitting during leisure-time, particularly television viewing time [8,9]. The 2011 American College of Sports Medicine Position Stand on Exercise Prescription recommends that, even for adults who meet physical activity and health guidelines, avoiding prolonged sitting should be a priority [10].

For most working adults, time spent sitting in the workplace is likely to be a greater contributor to overall sitting time than sitting during leisure-time [11]. As such, the workplace has recently been identified as a key setting in which to reduce adults’ sitting time to improve health [12,13]. Little data exists on the sedentary patterns of adults within the context of the workplace. In studies from The Netherlands [14] and Australia [11,15,16], workers have reported an average of three to five hours of sitting per day at work. However, such self-report estimates are likely to be subject to sizeable measurement error. The manner in which the estimates were derived also did not capture sedentary patterns across both workplace and non-workplace settings.

The use of small, unobtrusive electronic activity monitors (such as accelerometers) has made it possible to measure time spent sedentary and in physical activity with a greater level of precision and accuracy than has been possible with self-report alone [17]. As highlighted in two recent studies of office-based workers [18,19], estimates of workplace sitting are generally much higher when measured using devices (inclinometers), compared with estimates from studies that have relied on self-report. The use of accelerometers has also made it possible to measure time spent in light-intensity activities (typically walking time) during work hours. Light-intensity activity is difficult to capture via self-report, but may have important health implications for workers. Indeed, time spent in light-intensity activity has been shown to be beneficially associated with risk biomarkers for diabetes and heart disease [20-22]. While there is limited evidence on the effectiveness of workplace interventions to reduce sitting time in workers [23], recent interventions in overweight/obese adults [24] and older, non-working adults [25] have observed reductions in sedentary time, leading the authors to suggest that this change is facilitated through engagement in more light-intensity activities (e.g. standing, slow walking).

Accelerometers can measure not only total time spent at specific activity intensity but also the manner in which it is accumulated. Thus, they can be used to examine bouts of prolonged sedentary time. Prolonged sedentary time, reported as fewer breaks in sedentary time, has been shown to be detrimentally associated with several cardio-metabolic health outcomes [26,27]. Occupational health and safety guidelines recommend transitioning posture (e.g. from sitting to standing) at least every 30 minutes [28]. Moreover, a recent experimental study reported that interrupting sitting every 20 minutes was acutely linked to lower postprandial glucose and insulin levels – even if the interruptions were of light-intensity [29]. Office-based workers are particularly exposed to long periods of unbroken sitting during work hours [18]. However, it is not known whether differences in prolonged sedentary time exist between work hours and non-work hours. It is also unclear whether certain occupational groups are exposed to more prolonged sedentary time than others. To date, only one study has examined and reported significant differences in prolonged sedentary time across different subgroups of office-based workers [18].

Accelerometers enable activity patterns to be captured and compared not only within specific time frames (e.g. during work hours), but also across the whole day. In a study from the USA with 21 office-based workers [30], device-assessed sedentary time was shown to be significantly higher on work days compared to non-work days, despite no such difference being seen in physical activity. A recent study of Scottish postal workers [31] found that office-based workers spent more time sedentary during work hours and less time walking during non-work hours than did postal delivery workers. Further, their data clearly indicated postal delivery workers, spent significantly less time sedentary on work days compared with non-work days with less pronounced results for office workers. Whether these findings can be replicated in workers from other sedentary occupations remains to be determined, and further description of the patterns of workplace and non-workplace sedentary time (including both on work days and non-work days), across various occupations is warranted.

In a sample of Australian employees, we examined accelerometer-derived measures of sedentary time, sedentary time accrued in prolonged bouts (i.e. bouts ≥20 min and ≥30 min) and physical activity time in workplace and non-work contexts. Specifically, we compared these measures: 1) on work days versus non-work days; 2) during work hours versus non-work hours (on work days only); and, 3) across three workplace settings (office, call centre and customer service: during work hours only).

## Methods

### Participants

As previously described [32], four organisations based in Melbourne, Australia were approached to participate. From these four organisations, a convenience sample of 193 employees was recruited, via an internal email from human resources staff. To be eligible, participants were required to be ambulatory and to work a minimum of four days per week. Employees were from three workplace settings: office (n=131), call centre (n=36), and customer service (shop front claims processing; n=26). As described elsewhere [32] those participants who volunteered for the study were largely representative in both age and gender of the organisations from which they were recruited. The Ethics Committee of the Baker IDI Heart and Diabetes Institute approved the study and written informed consent was obtained from the organisations and employees involved.

### Procedures

The study was conducted over an eight-day period, with research staff visiting participants at their workplace on two separate days (day one, day eight). On day one, general demographic information (age, gender, education, marital status, and the nature of employment) was obtained via an interviewer-administered questionnaire. Weight and height were measured wearing light clothing and without shoes using a portable digital scale set with adjustable height rod (Charder Medical MS-3400 digital 300kg × 100g, Charder Electronic, Taiwan). Body mass index (BMI) was calculated as weight (kg)/height (m)^2^ and categorised to reflect weight status (normal or underweight <25 kg/m^2^; overweight 25–29.9 kg/m^2^; obese ≥30 kg/m^2^) [33].

### Assessment of sedentary and physical activity patterns

A uniaxial accelerometer (Actigraph GT1M, Pensacola, Florida, USA) was distributed to participants on day one and collected on day eight. The accelerometer was placed at the waist and secured by an elastic strap along the right anterior axillary line, and participants were requested to wear the accelerometer during all waking hours, except during water-based activities.

Actigraph accelerometers have been shown to provide a valid assessment of sedentary time [34,35] and physical activity [36] in free living adults. Accelerometer data were collected in one minute epochs. To accurately quantify work hours on work days, participants recorded the days on which they worked and the respective work start and finish times in a daily diary. Data were excluded from relevant analyses if work times were not reported (n=13 participants).

### Data processing

Accelerometer data were downloaded using ActiLife 3.2.2 software and summarized using SAS 9.2 for each relevant time period (e.g. work days, working hours, and hourly intervals). Non-wear periods were deleted for analyses; these were periods with at least 60 minutes of zero counts per minute (cpm), allowing for up to two consecutive, one minute interruptions (count values between 1–49 cpm) per non-wear period [37]. Data were also deleted for any relevant time period that included an activity count ≥20,000 cpm (n=2 days) [38]. Activity counts were categorised as sedentary (<100 cpm; predominantly sitting) [21], light-intensity activity (100-1951 cpm; typically gentle walking) [17], or MVPA (≥1952 cpm; typically at least brisk walking) [17]. Prolonged bouts of sedentary time were also identified, based on two definitions: ≥20 minutes, corresponding to definitions for which associations with clinical changes in cardio-metabolic biomarkers have been reported [29]; and, ≥30 minutes, corresponding to occupational health and safety guidelines [28]. Bouts were defined as starting and ending within the relevant time period (e.g. work hours), such that for a bout to be considered prolonged, at least 20 or 30 consecutive minutes of sedentary time needed to occur during the relevant period (e.g. work hours). Daily summaries of time spent sedentary, in prolonged sedentary bouts, and in light- and moderate activity for each time period were calculated in terms of the percentage of worn time spent at these intensities.

### Statistical analysis

Statistical analyses were performed in SAS 9.2; significance was set at p<0.05. Transformations were used to improve normality of some outcomes: the percentage worn time spent sedentary (inverse log), in light-intensity activity (log) and in MVPA (log [MVPA+0.001]). Analyses were by linear mixed models that accounted for repeated measures and adjusted for relevant confounders. Specifically, models that compared work with non-work days and work with non-work hours adjusted for workplace setting, while models that compared workplace settings adjusted for characteristics that varied across workplace settings (i.e. age, gender, education, marital status and BMI). Models were limited to valid days (wear time ≥10 hours, or wear time ≥75% of work hours for models that focused exclusively on time at work). Results from all models are reported as marginal means with 95% confidence intervals, back-transformed for any transformed variables.

To illustrate variation in activity patterns according to time of day, column graphs were created. These depict the number of minutes each hour of the day from 06:00 to 24:00 that was spent sedentary, in light-intensity activity and in MVPA, on work days and on non-work days. The column graphs only include data from valid days and from hours in which the accelerometer was not removed at all. The number of valid days on which each hour is based on is presented at the top of the column.

## Results

Table 1 shows that the majority of study participants were female (65.8%), of normal weight status (48.1%), had completed further education/attended university (77.0%) and worked in an office-based setting (70.1%). Participants from the three different workplace settings varied significantly in age (p=0.01), gender (p=0.03) and educational attainment (p<0.01). Participants from customer service settings tended to be similar to office employees in regards to these characteristics. However, call centre employees were significantly younger than office employees, were more likely to be male and were less likely to have completed a further education qualification than employees from the other settings. Call centre employees were also more likely to be obese and less likely to be married/living with their partner (defacto relationship) compared with customer service and office employees, though these differences did not reach statistical significance.

**Table 1:** **Characteristics of study participants** ^**a**^

	**All participants (n=187)**	**Office (n=131)**	**Call centre (n=33)**	**Customer service (n=23)**
Age, years; mean (SD)	37.3 (10.6)	38.3 (10.0)	32.3 (10.3)	38.5 (12.6)
Sex [Male]	64 (34.2%)	40 (30.5%)	8 (54.5%)	6 (26.1%)
Education (post-school qualification)	144 (77.0%)	109 (83.2%)	18 (54.5%)	17 (73.9%)
Marital Status (married or defacto)	126 (67.4%)	92 (70.2%)	18 (54.5%)	16 (69.6%)
Body Mass Index (BMI)				
Normal (BMI <25 kg/m^2^)	90 (48.1%)	64 (48.9%)	14 (42.4%)	12 (52.2%)
Overweight (BMI 25–29.9 kg/m^2^)	60 (32.1%)	45 (34.4%)	9 (27.3%)	6 (26.1%)
Obese (BMI >30 kg/m^2^)	37 (19.8%)	22 (16.8%)	10 (30.3%)	5 (21.7%)

^a^ Table includes all eligible participants (n=187) who participated in the self-report and accelerometer assessments.

Mean (SD) accelerometer wear time was 15.3 (1.8) hours on work days and 13.7 (2.0) hours on non-workdays. Significant differences between work and non-work days were observed for all outcome measures relating to physical activity and sedentary time (Table 2, columns 1 & 2). Compared with non-work days, work days involved proportionally more sedentary time along with less time in light-intensity activity and more time in MVPA. The proportion of time spent in prolonged sedentary bouts (i.e. bouts ≥20 min and ≥30 min) was also significantly higher on work days than on non-work days.

**Table 2:** **Sedentary time**, **prolonged sedentary bouts and physical activity of 180 employees during work and outside of work **^**a**^

	**All days**			**Work days only**		
	**Adjusted mean (95% CI)**		**p**	**Adjusted mean (95% CI)**		**p**
	**Non-work days**	**Work days**		**Non-work hours**	**Work hours**	
Number of days	345	758		737	738	
Mean wear time (hrs)	13.7	15.3		6.8	8.6	
**% of worn time spent**^**b**^						
All sedentary	62.9 (61.6, 64.1)	70.4 (69.5, 71.2)	<0.001	63.0 (62.2, 63.7)	77.0 (76.3, 77.6)	<0.001
Prolonged sedentary ≥20 min bouts	22.9 (21.1, 24.6)	29.6 (28.2, 31.1)	<0.001	23.8 (22.7, 24.9)	33.5 (32.1, 34.8)	<0.001
Prolonged sedentary ≥30 min bouts	14.4 (12.9, 16.0)	18.9 (17.6, 20.2)	<0.001	15.0 (14.0, 16.1)	21.5 (20.2, 22.7)	<0.001
Light -intensity physical activity	33.1 (31.9, 34.3)	25.0 (24.2, 25.8)	<0.001	30.3 (29.7, 31.0)	20.2 (19.6, 20.7)	<0.001
Moderate-vigorous physical activity	2.3 (2.1, 2.6)	3.7 (3.4, 4.9)	<0.001	4.3 (4.0, 4.7)	1.9 (1.7, 2.0)	<0.001

^a^ Table reports marginal mean and 95% CI from linear mixed models that account for repeated measures and adjust for workplace setting (office, customer service, call centre). Data were excluded for days with < 10 hours of wear (excludes n=13 participants).

^b^ Marginal means (95% CI) back-transformed for percentage worn time spent (sedentary [inverse log], in prolonged sedentary bouts of ≥20 minutes or ≥30 minutes, in light-intensity activity [log], and in MVPA (log of [MVPA % +0.001]).

Mean work time = 8.7 hours.

When sedentary and activity patterns on work days were compared during work and non-work hours (Table 2, columns 3 & 4), similar findings were observed to those reported for the work day versus non-work day comparisons (Table 2, columns 1 & 2). The only notable exception was work hours were comprised of proportionally less MVPA than non-work hours. Mean (SD) accelerometer wear time was 8.6 (1.3) hours during self-reported time at work and 6.8 (1.9) hours during self-reported non-work hours on those same work days. Most of participants’ work hours were spent sedentary (77.0%, i.e. 6.6 hours), with much of this time accrued in prolonged bouts of at least 20 or 30 minutes (i.e. 33.5% and 21.5% respectively of total work hours). The remaining time was comprised mostly of light-intensity activity (20.2%, 1.7 hours), with minimal MVPA (1.9%, 0.2 hours) recorded.

**Figure 1:**
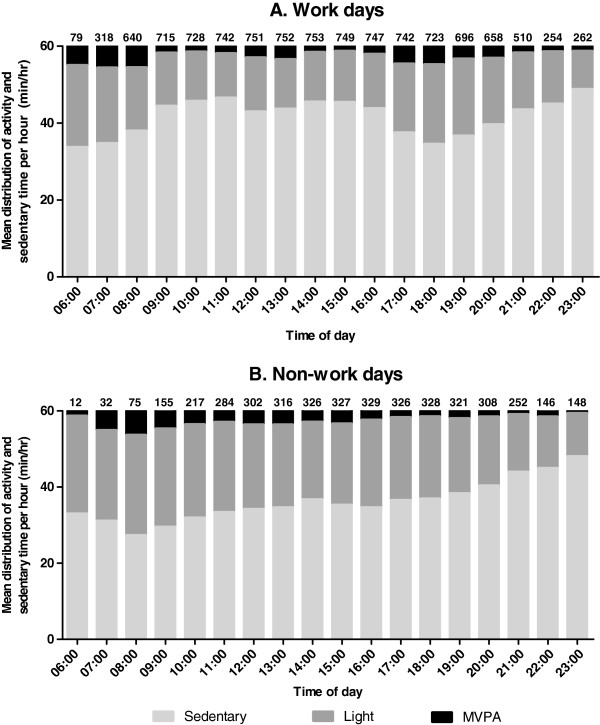
**Proportions of each daily hour from 06:00 to 22:00 spent sedentary, in light-intensity activity and MVPA on work days (Panel A) and non-workdays (Panel B).** Median work start time is 08:45; median work finish time is 17:17. **Footnote:** Values presented at the top of each column graph represent the number of valid days each one-hour period is based on.

A breakdown of the accelerometer output by hour of the day (Figure 1) illustrates the potential importance of time of day in these findings. On non-work days, greater sedentary time (and less physical activity) was observed as the day progressed, with the late evening hours involving the most sedentary time. On work days, late evenings also tended to be highly sedentary, and there was another highly sedentary period between 09:00–16:59 that coincided with the time when most people were at work (i.e. between the median work start [08:45] and finish times [17:17]). This same time period of 09:00–16:59 was when the most notable differences between work and non-work days with respect to the amount of time spent sedentary and in light-intensity activity was observed. The pattern of MVPA was distinct from the patterns of sedentary and light intensity time, with the most MVPA per hour being observed mostly outside of work hours at 07:00–08:59 on both work and non-work days and at 17:00–18:59 on work days, coinciding with the time period many participants were leaving work. The most pronounced differences in MVPA between work and non-work days were also mostly outside of working hours at 06:00–06:59 and 17:00–18:59.

The proportion of working time spent sedentary, in prolonged sedentary bouts, light-intensity activity and MVPA differed significantly across the three workplace settings (all p<0.001) (Table 3). Compared with customer service employees and office workers, call-centre employees spent proportionally more of their time at work sedentary and in prolonged sedentary bouts (≥20 and ≥30 mins), and less in light intensity activity and MVPA. Customer service workers spent significantly less of their time at work in prolonged sedentary bouts and more in light intensity activity than office workers. However, office workers spent proportionally more time at work in MVPA than customer service workers.

**Table 3:** **Sedentary time**, **prolonged sedentary bouts and physical activity for office** (**n**=**127**), **call centre** (**n**=**31**) **and customer service** (**n**=**23**) **employees during work hour **^**a**^

	**Adjusted mean (95% CI)**			**p**
	**Office**	**Call centre**	**Customer service**	
Number of work days	525	124	98	
Mean wear time (hrs)	8.8	8.2	7.5	
Mean work time (hrs)	8.9	8.2	7.5	
**% of worn time spent **^**b**^				
All sedentary	75.8 (74.5, 77.1)	83.4 (81.3, 85.2) †	73.7 (70.2, 76.8) ‡	<.0001
Prolonged sedentary, ≥20 min bouts	33.4 (30.8, 36.0)	42.5 (36.8, 48.2) †	20.6 (14.5, 26.7) †‡	<0.001
Prolonged sedentary, ≥30 min bouts	21.4 (19.0, 23.7)	29.7 (24.6, 34.7) †	9.6 (4.3, 15.0) †‡	<0.001
Light -intensity physical activity	20.6 (19.5, 21.8)	15.3 (13.6, 17.3) †	24.4 (21.5, 27.8) †‡	<.0001
Moderate-vigorous physical activity	2.4 (1.9, 2.8)	0.7 (0.4, 0.9) †	1.3 (0.8, 1.7) †‡	<.0001

† Different to office employees (p<0.05) ‡ Different to call centre employees (p<0.05).

^a^ Table of marginal means (95% CI) from linear mixed models that account for repeated measures and adjust for age (years), gender, BMI (overweight or obese: yes/no), marital status (married or defacto: yes/no), education (post school qualification: yes/no). Data were excluded for days with <75% of time at work unobserved (n=20 days) and for participants without self-reported covariate data (n=13 participants).

^b^ Marginal means (95% CI) back-transformed for percentage worn time spent (sedentary [inverse log], in prolonged sedentary bouts of ≥20 minutes or ≥30 minutes, in light-intensity activity [log], and in MVPA (log of [MVPA % +0.001].

## Discussion

It is well recognized that the workplace is an important setting for physical activity promotion initiatives [39]. This study highlights that it is also a key setting for addressing prolonged sedentary time – an independent risk factor for early death and poor health outcomes. In this sample of Australian employees, sedentary time (derived from accelerometers) comprised more than three-quarters of total work hours and work time also involved a considerable amount of sedentary time that was accrued in prolonged bouts (≥20 or ≥30 minutes), particularly within call-centre employees. Furthermore, the differences that were observed between work days and non-work days (where employees spent a significantly greater proportion of time sedentary and in prolonged sedentary bouts, and proportionately less in light-intensity activity) appeared to be attributable primarily to the differences between work and non-work hours.

Our findings are similar to those of a recent observational study from the USA of 21 desk-bound workers [30], which found that sitting, assessed using a monitor that incorporates both inclinometers and accelerometers, was higher on workdays than non-work days by 110 minutes per day. The reported difference, equating to approximately 9.9%, was similar to our observed 7.5% difference between work and non-work days.

The observation that employees spent proportionally more time in MVPA on work days compared with non-work days is consistent with several studies [30,31] that have reported a tendency for sedentary office employees to engage in at least as much physical activity (as measured by step count) on work days as non-work days.

Our analysis of the accelerometer output by hour of the day suggests that differences observed between work days and non-work days are strongly influenced by sedentary and activity patterns during employees’ work hours. Specifically, the time period of 09:00–16:59, when many participants were working, was when significant differences in both sedentary time and time spent in light-intensity activity were observed between work days and non-work days. By contrast, the time periods of 06:00–06:59 and 17:00–18:59 which are typically outside of usual working hours was when MVPA was significantly higher on work days than non-work days. The occurrence of additional MVPA on work days, outside of work hours, may explain why work days, but not work hours involved proportionally more MVPA than non-working days. Additional data collection on how employees’ activity was accumulated, for example, in the form of structured exercising after work or via active transportation could yield important insights relevant to promoting activity with time- and context- specific interventions such as mobile text messaging.

In our pooled study sample, employees were sedentary for an average of 6.6 hours while at the workplace. This is considerably higher than figures based on self-report in Dutch full-time workers (2.7 hours inclusive of work-related travel) [14] and Australian workers in professional and white collar occupations (3.5 hours and 4.1 hours, respectively) [15]. Our observation that approximately three quarters of work time was spent sedentary is comparable to a recent study of 140 Swedish call-centre operators that used inclinometers (mean 77% vs 75% respectively) [19]. While most self-report studies examining workplace sitting time do not report on employees start and finish work times [11,14-16], there is evidence to suggest that the high sedentary time seen in our study (when reported as a proportion of work hours) is not entirely due to our sample of employees reporting longer than normal work hours. Professional and white collar workers have been reported to spend an average of 81% of their working hours performing activities of light-intensity or lower [16]; in the present study, the combined averages for light-intensity (22%) and sedentary time (77%) were 97%. Caution is warranted in making direct comparisons between the studies, as employees were recruited from different occupational groups.

In addition to our sample of workers spending the majority of time at work sedentary, we observed that nearly half of sedentary time at work was accrued in prolonged sedentary bouts of at least 20 or 30 minutes (i.e., 33.5% and 21.5% respectively of total work hours). To date, only one other study has objectively quantified the sitting patterns of office-based workers across an assumed typical work day (9:00–17:00) [18]. This study, which used the ActivPal activity monitor on 83 office workers, found an even higher proportion of sitting time at work (52% and 67%) was accrued in bouts of prolonged sitting that lasted longer than 20 or 30 minutes in duration, respectively.

Our study adds to a growing body of evidence suggesting that the workplace is a key setting for sedentary behaviours. Additional studies are needed to support these findings and to further explore the potential impact of sedentary behaviour at work on activity patterns outside of work. Such information is needed to inform public health guidelines aimed at reducing sedentary time, particularly those strategies that might target the workplace setting. These studies need high-quality assessment of behaviour that occurs during employees actual working hours, e.g. as reported in diaries, rather than assumed working periods of eight hours duration [31]. This will help to avoid potentially substantial misclassification that might occur from MVPA occurring immediately after work, but within these assumed working time periods.

A novel element of our study was the examination of accelerometer-derived sedentary time and physical activity across different workplace settings. Here, variations across the three workplace settings selected were considerable. Not surprisingly, call centre workers were the most sedentary and least physically active during work hours. Call centre employees also accrued more of their sedentary time through prolonged bouts. In contrast, customer service employees had the lowest levels of prolonged sedentary time. These differences likely reflect variations in the opportunities of employees to interrupt sedentary periods through task-based activities. For employees working in settings that afford little or no task-based opportunities to interrupt sedentary time (such as call-centres), alternative options for reducing workplace sedentary time may be required. For example, introducing sit-to-stand workstations into call centres could provide employees with additional opportunities to interrupt prolonged sedentary time by enabling them to transition from sitting to standing and work in an upright posture intermittently throughout the work day.

A strength of our study was the combined use of an accelerometer and a self-report daily diary to provide individually tailored segmentation of sedentary and physical activity time occurring during work and non-work hours on work days. A key limitation of the accelerometer was that it was not able to provide a postural assessment of sitting, which is possible with other activity monitors such as the ActivPAL device [40,41]; thus some time spent standing still may have been classed as sedentary time. In this study, a widely used cut point of <100 cpm was selected to estimate sedentary time. Whilst this is considered adequate for use in adult studies [26,36,42] there is no universally accepted cut point and other cut points, e.g. <150 cpm [43], have also been advocated. Similarly, light and MVPA were estimated using the well regarded Freedson cut points [17], although these are also not universally accepted. The selection of a different epoch length, different cut-points, or criteria for determining a bout of prolonged sedentary time would have led to different estimates of the mean amount of each activity and prolonged sedentary time. However, as this would be likely to occur equally for all days and times, our comparisons between work and non-work are unlikely to be affected. As is often the case in such studies, wear time did not appear to cover all waking hours and was estimated indirectly rather than observed, which may have affected results, since more non-wear time tended to be detected outside of work hours than during work hours, and on non-work than work days. Other limitations include our use of a convenience sample, and low numbers of call centre and customer service employees, limiting generalizability to the broader working population. Studies that use more sophisticated technologies cable of differentiating between standing and sitting time, along with probabilistic sampling covering a broader spectrum of employee and job attributes, are needed to better assess sedentary and physical activity time in the workforce and understand differences across workplace settings.

## Conclusions

We found in this sample of Australian employees that the majority of work hours were spent sedentary, and that work time involved a substantial proportion of time spent in prolonged sedentary bouts. Sedentary and light-intensity physical activity time accumulated during employee’s work hours were the main basis for the differences observed between work and non-work days. There is emerging evidence that sedentary time is associated with increased risk of major chronic diseases [20] and that breaking up sedentary time is beneficially associated with markers of cardio-metabolic health [26,27]. Our findings suggest the need for further device-based measurement studies in workplaces, and for clarifying the potential health risk for workers who are exposed to prolonged periods of unbroken sitting. As the relevant evidence accumulates, it is prudent that future workplace health-promotion initiatives should not only be aimed at increasing physical activity participation, but also reducing and breaking up prolonged sedentary time. Workplaces such as call centres, in which prolonged sedentary time is especially high and physical activity is low, may require particular attention.

## Abbreviations

MVPA: Moderate-to-vigorous physical activity; Cpm: Counts per minute.

## Competing interests

The authors declare that they have no competing interests.

## Authors’ contributions

All authors made substantial contributions to the conception and design of the study. AT was involved in the acquisition of data. AT, GH and EW drafted the manuscript and NO, DD, PG and BC revised it critically for important intellectual content. AT, GH and EW performed the statistical analyses. All authors were responsible for the interpretation of the data. All authors read and approved the final manuscript.
